# PCR and serology find no association between xenotropic murine leukemia virus-related virus (XMRV) and autism

**DOI:** 10.1186/2040-2392-1-14

**Published:** 2010-10-14

**Authors:** Brent C Satterfield, Rebecca A Garcia, Fiorella Gurrieri, Charles E Schwartz

**Affiliations:** 1 Cooperative Diagnostics, LLC, Greenwood, SC 29646, USA; 2 Institute of Medical Genetics, Catholic University, Rome, Italy; 3 Greenwood Genetic Center, Greenwood, SC 29646, USA

## Abstract

Xenotropic murine leukemia virus-related virus (XMRV) is a retrovirus implicated in prostate cancer and chronic fatigue syndrome (CFS). Press releases have suggested that it could contribute to autism spectrum disorder (ASD). In this study we used two PCR assays and one antibody assay to screen 25 blood samples from autistic children born to mothers with CFS and from 20 mixed controls including family members of the children assayed, people with fibromyalgia and people with chronic Lyme disease. Using a real-time PCR assay, we screened an additional 48 South Carolina autism disorder samples, 96 Italian ASD samples, 61 South Carolina ASD samples and 184 healthy controls. Despite having the ability to detect low copy number XMRV DNA in a large background of cellular DNA, none of the PCR assays found any evidence of XMRV infection in blood cells from patients or controls. Further, no anti-XMRV antibodies were detected, ruling out possible low level or abortive infections in blood or in other reservoirs. These results imply that XMRV is not associated with autism.

## Findings

Xenotropic murine leukemia virus-related virus (XMRV) is a recently discovered retrovirus that can infect humans [1]. Studies of the virus in prostate cancer have given conflicting results, with between 0% and 27% of prostate cancers suggested to be associated with XMRV [2-4]. More recently, Lombardi, *et al *showed that 67% of people with chronic fatigue syndrome (CFS) were positive for XMRV by PCR amplification of the *gag *gene [5]. In an interview given on the same day as the Lombardi publication, Dr Mikovits stated that they had found XMRV in a 'significant number' of autism spectrum disorder (ASD) samples and speculated that 'this might even explain why vaccines lead to autism in some children' [6]. Shortly thereafter, widely circulated articles appeared, containing non-peer reviewed data with reports that XMRV may be present in ≥40% of people with autism [7]. Given the recent controversy over the connection between ASD and the MMR (measles, mumps, rubella) vaccine, a scientific evaluation of these statements is important [8,9]. ASD comprises multiple cognitive and developmental disorders, including autistic disorder (AD), Asperger disorder and PDD-NOS (pervasive developmental disorder, not otherwise specified). Although ASD affects an estimated 1 in every 110 individuals, no consistent causes for the disease have been identified [10].

In this study, we screened for a link between XMRV and autism. First, we looked for XMRV in 25 blood samples from children with ASD born to mothers with CFS and in 20 samples from a mix of controls including healthy family members of the children, people with fibromyalgia and people with chronic Lyme disease. We also assayed genomic DNA from 96 Italian ASD samples, 48 South Carolina AD samples, 61 South Carolina ASD samples and 184 healthy controls for presence of XMRV DNA. The healthy controls were a mix of healthy male and female college students and newborns from South Carolina.

A novel real-time PCR assay targeting the *pol *gene in murine leukemia virus (MuLV) and XMRV was used to detect XMRV (Table 1). The primers and probes for the assay were designed against regions of XMRV that were 100% conserved in the seven sequenced XMRV strains (available in GenBank; accession numbers GQ497344.1, GQ497343.1, NC_007815.1, EF185282.1, DQ399707.1, DQ241302.1, DQ241301.1). The *pol *assay targets a conserved region in murine leukemia viruses, allowing the detection of potentially mutant strains of XMRV. Sensitivity of the assay was determined by running dilutions (600, 60 and 6 copies per reaction) of synthetic DNA of the VP62 XMRV strain in a background of 2.5 μg of genomic DNA. DNA from XMRV-infected 22Rv1 cells was used as an additional validation of the sensitivity of each assay [11]. Dilutions of 1000, 100 and 10 pg were used in a background of 2.5 μg of genomic DNA. Assuming 6 pg/cell, these dilutions correspond to 170, 17 and 1.7 infected cells per reaction.

**Table 1:** Real-time PCR primer and probe sequences for the *pol *xenotropic murine leukemia virus-related virus (XMRV) assay.

Name	**Sequence (5'→3')**^**1**^
*pol *(2794 to 3062)	
Primers	
Forward	GGGGATCAAGCCCCACATA
Reverse	GGTGGAGTCTCAGGCAGAAAA
Probe	[6FAM] TGTTCCAGGGGGACTGGCAAGGTACCAccctgg [DABC]^2,3^

^1^Reference sequence was the VP62 XMRV strain (GenBank: EF185282.1).

^2^Lower case bases were added to form the stem.

^3^[6FAM] and [DABC] are the fluorophore FAM and the quencher Dabcyl, respectively.

Samples were obtained from the autistic children of mothers with CFS and from banked, well-characterized autism samples at the Greenwood Genetic Center. Informed consent was obtained from all participants or legally authorized representatives and identifying information was removed from each sample. For the former cohort, blood was collected, and the buffy coat was immediately isolated for nucleic acid extraction (Blood DNA Minikit; Qiagen, Valencia, CA, USA). Plasma samples were frozen for later analysis. Extracted DNA was quantified on a spectrophotometer (Nanodrop; Thermo Scientific, Wilmington, DE, USA) and checked for integrity with a minimum 260:280 ratio of 1.8. DNA was diluted to 100 ng/μl. For the latter cohort and the healthy controls, DNA was extracted from blood using proteinase K lysis and high-salt precipitation as previously described [12]. For the samples from autistic children born to mothers with CFS, 25 μl of sample (2.5 μg of DNA and 833 ng of peripheral blood mononuclear cell (PBMC) DNA) were added to 25 μl of a commercial master mix (Simplex DNA Master Mix; Cooperative Diagnostics, Greenwood, SC, USA) with a final primer concentration of 250 nmol/l and probe concentration of 200 nmol/l. The 25 ASD samples from children born to mothers with CFS and the 20 mixed control samples were also submitted to the Centers for Disease Control (CDC) for blinded assaying using an XMRV western blot and a nested PCR assay (external primers XPOLOF and XPOLOR; internal primers XPOLIF and XPOLIR as shown in the Switzer, et al publication) that detects both MuLV and XMRV *pol *gene sequences [13]. The CDC nested PCR assay used 1.0 μg of DNA and was capable of detecting < 10 copies of VP62 XMRV DNA. The DNA integrity of the samples was verified by β-actin PCR as an internal control, as described previously [13]. The western blot used denatured XMRV as antigen, which was crossreactive with anti-MuLV-positive goat sera for the envelope (glycoprotein (gp)69/71), transmembrane (p15E), matrix (p15), Gag (pr68) and capsid (p30) antigens. For the banked samples and healthy controls, 5 μl of sample (500 ng of DNA and 167 ng of PBMC DNA) were added to 5 μl of a master mix (Simplex DNA Master Mix; Cooperative Diagnostics) with primer and probe. Real-time PCR cycling conditions were 95°C for 20 seconds followed by 45 cycles of 95°C for 1 second and 60°C for 20 seconds. Two positive controls (10 pg of XMRV infected 22Rv1 cell DNA or 60 copies of VP62 XMRV synthetic DNA spiked into 2.5 μg of genomic DNA) and two negative controls (nuclease-free water) were included in each run.

**Figure 1:**
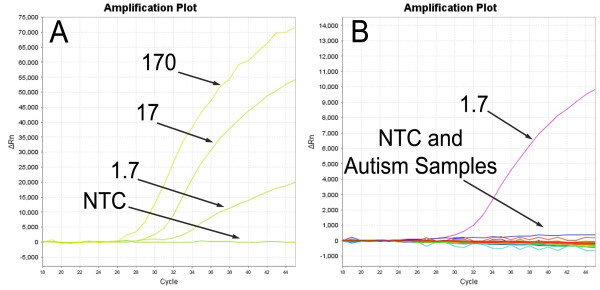
**Absence of Xenotropic murine leukemia virus-related virus (XMRV) in autism using real-time PCR**. **(A) **XMRV *pol *gene detection of dilutions of XMRV DNA extracted from infected 22Rv1 cells (170, 17, 1.7 and 0 cells, assuming 6 pg DNA per cell) in a background of 2.5 μg DNA. **(B) **Typical results for autism samples using *pol *gene real-time PCR with a positive control of XMRV DNA from 1.7 infected 22Rv1 cells in a background of 2.5 μg DNA. There were no positives other than the positive control.

The *pol *real-time PCR assay was able to detect XMRV DNA extracted from infected 22Rv1 cells in a background of 2.5 μg DNA (833 ng PBMC DNA), increasing the sensitivity 10 to 25 times if that described by Lombardi *et al. *(Figure 1) [5]. The CDC PCR assay was capable of detecting < 10 copies of XMRV in a background of 1 μg genomic DNA. However, after analyzing the samples from 25 autistic children born to mothers with CFS and 20 mixed controls, there was no evidence of XMRV infection in any of the PCR assays (Table 2). No anti-XMRV antibodies were detected in these samples either, ruling out low-level infections that might have been missed by PCR. Additional assaying of AD, ASD and healthy control samples by real-time PCR revealed no evidence of XMRV infection. In all, 230 autism samples and 204 controls gave negative PCR results for XMRV.

**Table 2:** Results of xenotropic murine leukemia virus-related virus (XMRV) real-time PCR.

Sample	Positives by assay^1^		
	*pol*	CDC^2 ^*pol*	WB
Autistic children of mothers with CFS^3^	0/25	0/24	0/25
Mixed controls	0/20	0/19	0/20
Italian ASD^4^	0/96		
South Carolina autism disorder	0/48		
South Carolina ASD	0/61		
Newborn healthy controls	0/96		
Male healthy controls	0/88		

**Totals**	**0/434**	**0/43**	**0/45**

^1^Shows number of samples assayed and number of positives, including results for *pol*, CDC *pol *genes and a western blot (WB) for detection of anti-XMRV antibodies.

^2^CDC, Centers for Disease Control.

^3^CFS, chronic fatigue syndrome.

^4^ASD, autism spectrum disorder.

In detection of any new virus, false positives and false negatives are always a concern. In this case, the ability to detect low copy numbers of XMRV by two different PCR assays, both designed to recognize regions of XMRV/MuLV that are 100% conserved in all published XMRV strains, implies that the assays would be able to detect XMRV if it was present in the samples. It is possible that XMRV was present in the samples at levels below the detection limit of the PCR assays, but this seems unlikely given the relatively high frequency of infection found by Lombardi *et al. *in people with CFS using a much smaller amount of sample [5]. The samples could have inhibited the PCR assay, but this is unlikely as XMRV DNA spiked into random patient samples was readily detected in every case. Further, no anti-XMRV antibodies were detected, precluding the possibility of low-level or abortive infections in blood or other reservoirs that the PCR assay might have missed.

The presence of false positives in the CFS-XMRV findings cannot be ruled out, and it is supported by the following observations. A previous attempt to validate the findings of Lombardi *et al. *[5] found no XMRV in 186 CFS samples [14]. A second independent attempt also failed to find any association between XMRV and CFS, using both PCR and XMRV antibody assays in the 170 CFS samples assayed [15]. A third attempt in the Netherlands also failed to find XMRV in 32 CFS samples [16], and a fourth attempt in the US also failed to find XMRV in 51 CFS samples [13].

Another interesting differentiator between our data and previous results is the lack of evidence of PCR-detectable XMRV in the blood of healthy controls. The Lombardi *et al. *study showed that in addition to 67% of people with CFS having PCR-detectable levels of XMRV in the blood, there may be a baseline of infection within the general population that is as high as 4% [5]. The three European studies, all of which failed to find evidence of XMRV in the blood of controls or CFS samples, seem to suggest that the 4% rate detected by Lombardi *et al. *is much greater than the actual prevalence in the world, and may have been inflated by either geographically confined assaying or false positives [14-16]. Our results confirm that suggestion; we found no evidence of XMRV in 204 controls and 230 autism samples (434 samples in total).

From the results presented here, if XMRV is present in people with autism, it is an extremely rare occurrence and is unlikely to be a significant contributor to the disorder.

## Competing interests

BCS and RAG are employees of Cooperative Diagnostics. Cooperative Diagnostics is a commercial enterprise that owns the rights to the XMRV real-time PCR assay described in this manuscript, in addition to the Master Mix that was used. Publication of these results may well reduce the potential market that Cooperative Diagnostics could reach with its XMRV assay.

## Authors' contributions

BCS and RAG designed the XMRV assay. CES and BCS planned the experiments, and RAG ran the assays. BCS wrote the paper. CES and FG contributed samples and expertise surrounding autism.
